# Cognition and Cognitive Reserve in Cochlear Implant Recipients

**DOI:** 10.3389/fnagi.2022.838214

**Published:** 2022-03-21

**Authors:** Christiane Völter, Lisa Götze, Marcel Bajewski, Stefan Dazert, Jan Peter Thomas

**Affiliations:** ^1^Department of Otorhinolaryngology, Head and Neck Surgery, Catholic Hospital Bochum, Bochum, Germany; ^2^Department of Otorhinolaryngology, Head and Neck Surgery, St.-Johannes-Hospital, Dortmund, Germany

**Keywords:** hearing loss, cognition, cochlear implantation, cognitive reserve, depression, plasticity

## Abstract

At present, dementia is a hot topic. Hearing loss is considered to be a modifiable risk factor for cognitive decline. The underlying mechanism remains unclear and might be mediated by socioeconomic and psychosocial factors. Cochlear implantation has been shown not only to restore auditory abilities, but also to decrease mental distress and to improve cognitive functions in people with severe hearing impairment. However, the promising results need to be confirmed. In a prospective single-center study, we tested the neurocognitive abilities of a large group of 71 subjects with bilateral severe hearing impairment with a mean age of 66.03 (SD = 9.15) preoperatively and 6, 12, and 24 months after cochlear implantation using a comprehensive non-auditory computer-based test battery, and we also assessed the cognitive reserve (CR) [Cognitive Reserve Index (CRI)], health-related quality of life (QoL) (Nijmegen Cochlear Implant Questionnaire), and depression (Geriatric Depression Scale-15). Cognitive functions significantly increased after 6 months in attention (*p* = 0.00004), working memory (operation span task; *p* = 0.002), and inhibition (*p* = 0.0002); and after 12 months in recall (*p* = 0.003) and verbal fluency (*p* = 0.0048), and remained stable up to 24 months (*p* ≥ 0.06). The CR positively correlated with cognitive functions pre- and post-operatively (both *p* < 0.005), but postoperative improvement in cognition was better in subjects with poor CR (*p* = 0.003). Depression had only a slight influence on one subtest. No correlation was found among cognitive skills, quality of life, and speech perception (each *p* ≥ 0.05). Cochlear implantation creates an enriched environment stimulating the plasticity of the brain with a global positive impact on neurocognitive functions, especially in subjects with poor preoperative cognitive performance and low cognitive reserve.

## Introduction

Due to the aging of the society, the number of subjects suffering from dementia has increased to about 55 million worldwide today and it will be rising up to 78 million by 2030 and even 139 million by 2050 (WHO, 2021b). Cognitive decline begins from middle age onward, and it particularly affects fluid intelligence (executive functions, processing speed, attention, and episodic memory), while crystalline intelligence (implicit knowledge and semantic memory) is not affected by aging (Salthouse, 2010). Despite comprehensive efforts in this field, there is no treatment available to cure or slow down the progress of the disease (Perneczky et al., 2019). Therefore, research mainly focuses on dementia prevention by reducing modifiable risk factors, such as excessive alcohol consumption, head injury, air pollution, poor education, hypertension, smoking, obesity, depression, physical inactivity, diabetes, infrequent social contacts, and hearing loss. Adequate treatment of hearing impairment in midlife is suggested to decrease the prevalence of dementia by 8% (Li et al., 2019; Livingston et al., 2020).

Like cognitive decline, the prevalence of hearing impairment also increases with age; currently, 430 million people are affected by hearing loss disability (Tran et al., 2021; WHO, 2021a). Hearing loss can negatively impact both the physical and also the psychological well-being of the patients and their partners (Scarinci et al., 2009; Jiam et al., 2016; Deal et al., 2019; Völter et al., 2021a). A meta-analysis done by Lawrence et al. in 2020 revealed a significant association between hearing loss and depression in cross-sectional and longitudinal studies with an odds ratio of 1.54 and 1.39, respectively (Lawrence et al., 2020). Women aged from 60 to 69 with an untreated hearing impairment suffer more frequently from social isolation than men (Mick and Pichora-Fuller, 2016; Shukla et al., 2020). Furthermore, Lin and colleagues demonstrated in a study on 2017 individuals that persons with a hearing loss of at least 25 dB also have a 1.4-fold elevated risk of falling for every 10 dB of increase in hearing loss (Lin and Ferrucci, 2012) as recently confirmed by a meta-analysis even after adjustment for other risk factors (Jiam et al., 2016).

The close interaction between hearing loss and cognitive functions has gained attention of researchers recently (Panza et al., 2019; Powell et al., 2021). On the one hand, neurocognitive functions have an impact on speech understanding, especially in challenging acoustic situations (Rönnberg et al., 2013; Völter et al., 2020a,b); on the other hand, several studies have pointed out the negative impact of hearing loss on cognition, both on a behavioral as well as on a neuroanatomic level (Deal et al., 2015; Armstrong et al., 2020; Manno et al., 2021; Ren et al., 2021; Völter et al., 2021a). A longitudinal study conducted by Lin in 2011 demonstrated that the risk of dementia within 11.9 years was elevated by 1.89 times in people with mild-hearing impairment, 3-fold in people with moderate hearing impairment, and by 4.94 times in people with severe hearing impairment (Lin et al., 2011). This observation was underlined in a recent meta-analysis on 36 studies describing a statistically significant correlation between age-associated hearing impairment and dysfunction in global cognition (Loughrey et al., 2018). Listening effort and cognitive load caused by hearing loss are proposed to be the underlying mechanisms between the association between peripheral and central level processing disorders (Peelle, 2018; Uchida et al., 2019; Slade et al., 2020).

Various options are available to treat hearing loss, ranging from fitting conventional hearing aids to operative middle ear procedures, bone conduction implants, active middle-ear implants, and cochlear implants (CI) (Löhler et al., 2019). A CI is an electronic inner ear prosthetic device that bypasses the hair cells in the cochlear region by directly stimulating the auditory nerve (Lenarz, 2018; Dazert et al., 2020). Today, cochlear implantation is a well-established approach to restore hearing in subjects with severe to profound hearing loss with little benefit from conventional hearing aids (Boisvert et al., 2020; Carlson, 2020; Dazert et al., 2020). In most cases, significant improvements in speech understanding can be obtained within the first 6 months after implantation, although improvements up to or beyond 2 years are also reported (Lenarz et al., 2012; Kelsall et al., 2021).

Furthermore, positive outcomes of auditory rehabilitation with regard to the quality of life (QoL), psychosocial comorbidities, and cognitive functions have been reported (Olze et al., 2011; Völter et al., 2018; Häußler et al., 2019; Andries et al., 2021). Providing subjects with hearing loss with an adequate sensory input is supposed to help to reduce the cognitive load caused by hearing loss and to release other cognitive resources. Therefore, the question arose whether auditory rehabilitation by cochlear implantation can counteract dementia in the long term. This was first studied by Mosnier in 2015 in a multicenter study, and since then, there has been a growing number of publications dealing with this topic in the last decade (Jayakody et al., 2017; Mosnier et al., 2018; Sarant et al., 2019; Huber et al., 2020; Mertens et al., 2021; Völter et al., 2021a).

Despite promising results, the data are still conflicting not only due to the close association between hearing and cognition which influence each other in different ways, but also because of the study designs used (Dawes, 2019; Moberly et al., 2019). Some protocols used auditory-based neurocognitive assessments, and therefore, limited understanding of the test material by subjects with severe hearing impairment cannot be ruled out (Pye et al., 2017; Dawes, 2019; Utoomprurkporn et al., 2020; Völter et al., 2020b, 2021b; Mertens et al., 2021; Raymond et al., 2021). Others only applied screening test instruments which might not be sensitive enough to detect slight differences (Sonnet et al., 2017; Gurgel et al., 2021). In general, the sample size is often small and the follow-up period is short (Cosetti et al., 2016; Gurgel et al., 2021). Further, some only used qualitative methods for the evaluation of cognitive improvement, while others used multiple cognitive measures without correcting the multiple comparisons (Mosnier et al., 2015; Cosetti et al., 2016; Ambert-Dahan et al., 2017; Völter et al., 2018; Gurgel et al., 2021). Few studies repeatedly evaluated speech, cognitive domains, and health-related QoL of the same participants to study the impact of auditory restoration on cognition (Mosnier et al., 2015; Völter et al., 2018, 2021a).

In general, multiple factors might contribute to the cognitive performance in the elderly. Cognitive reserve (CR) is a modifiable factor in cognitive decline and has recently been added to the life-course model of possible modifiable risk factors for dementia (Livingston et al., 2020). The term of CR is a latent construct which was introduced by Stern as a mechanism to explain individual differences in rates of cognitive decline as in up to 33% of cognitively healthy elderly people (assessed by clinical measures before death) full pathologic criteria for Alzheimer’s disease in post-mortem investigation were found (Ince, 2001). The CR is mostly studied not only on subjects with dementia (Lavrencic et al., 2016), but also on subjects suffering from other brain pathologies, such as stroke (Rosenich et al., 2020), multiple sclerosis (Sumowski et al., 2014), Parkinson’s disease (Guzzetti et al., 2019), or traumatic brain injury (Stenberg et al., 2020). So far, CR has been defined and used in different ways (Pettigrew and Soldan, 2019). According to a recent publication by Stern in 2020, CR refers to the adaptability of cognitive processes that help explain the differential susceptibility of cognitive abilities to brain aging (Stern et al., 2020).

Cognitive reserve cannot be directly measured, and is mostly indexed by education as it is well known that older adults with a higher level of education showed better global and detailed neuropsychological function than those with lower educational levels (Wilson et al., 2019). However, numerous studies have demonstrated that education only partially contributes to CR and its contribution is limited to its association with the level of cognitive functions before old age (Clare et al., 2017). Gow et al. demonstrated in a large longitudinal study that engagement in leisure activities in midlife is positively associated with cognitive abilities (Gow et al., 2017). Chan et al. underlined this by analyzing a cohort of 205 individuals (aged 66 to 88 years), from the Cambridge Center for Ageing and Neuroscience (Chan et al., 2018). Lifestyle activities in midlife significantly contributed to cognitive ability in late-life, independent of education and occupation. This has also been described in patients with either subjective memory decline or those who were diagnosed with a mild cognitive impairment (MCI) (Lee et al., 2020). Whereas the educational background was not correlated with a comprehensive cognitive assessment, leisure activities were mostly predictive for executive function and visuospatial abilities. Therefore, multiple indicators, such as occupational attainment and stimulating leisure activities (reading, playing games, playing music, and social activities) have to be combined to assess CR (Opdebeeck et al., 2016). One of the most common assessments is the Cognitive Reserve Index Questionnaire (CRIq), primarily introduced by Nucci in 2012 (Nucci et al., 2012).

Recently, the importance of leisure activities and occupational attainment on cognitive functions has also been recognized in people with hearing impairment (Evans et al., 2018; Chen and Lu, 2020; Gao et al., 2020; Chen, 2021). In the Chinese Longitudinal Healthy Longevity Survey, a longitudinal study on 6,309 elderly, who were aged 65 years, it was found that the CR increased the odds ratio of cognitive impairment in people with hearing impairment from 1.4 in case of a high CR to 2.59 with a middle CR and even to 4.32 for those with a low CR, in contrast to 1.66 in normal-hearing counterparts (Chen and Lu, 2020).

Due to the social and economic importance of this topic, the aim of the present prospective study was to add more data to the ongoing discussion. Therefore, we analyzed in a large population with a comprehensive non-auditory based neurocognitive assessment, whether (1) auditory rehabilitation by cochlear implantation can lead to improvements in cognitive functioning in the long-term follow-up of 24 months, and (2) whether cognitive reserve and psychosocial factors, such as depression and self-reported quality of life, impact the cognitive functioning.

## Materials and Methods

### Participants/Study Samples

Inclusion criteria were defined as follows: (1) Postlingual hearing impaired, (2) aged more than or equal to 50 years, (3) suffering from a severe to profound bilateral hearing loss with a 4-PTA (average of the frequencies 0.5, 1, 2, and 4 kHz) greater than or equal to 61 dB, (4) sufficient knowledge of the German language, (5) absence of a global cognitive impairment as assessed by the Mehrfachwahl-Wortschatz-Intelligenztest-B (MWT-B) (Lehrl, 2005), (6) free from central nervous system disease or treatment with anticholinergic medication, and (7) good (or corrected) near vision.

About 101 consecutive CI candidates presented at the ENT Department of the University of Bochum and scheduled for cochlear implantation between 2016 and 2018 were enrolled. The CI candidacy was determined according to the German guidelines for cochlear implantation which requires the best aided monosyllabic speech understanding of less than 60% at 65 dB (Dazert et al., 2020). Implantation was performed on people with poor hearing ability. None of the patients were provided with an electro-acoustic system (EAS). Fifty-four patients had a hearing device and 14 patients had a CI on the contralateral ear. Fifteen patients had to be excluded due to visual impairment (*n* = 4), upper limb motor dysfunction (*n* = 1), language barrier (*n* = 8), and 2 due to the onset of psychiatric disorder. Eighty-six people underwent cognitive testing prior to cochlear implantation [mean age, 67.89 (SD = 8.9)]. Seventy-one of the 86 subjects [mean age, 66.03 (SD = 9.15)] performed cognitive assessment prior to as well as 12 and 24 months post cochlear implantation whereas 15 of the 86 subjects initially dropped out during the 24 months due to serious health problems (*n* = 4), death (*n* = 1), unwillingness to participate (*n* = 6), or relocation (*n* = 4). Sixty-seven of the 71 subjects underwent additional cognitive assessment after 6 months.

Data on the 71 subjects included in the present study are summarized in Table 1.

**TABLE 1:** Demographic and audiometric data prior to implantation standard deviation (SD); 4-pure-tone average (PTA); sound pressure level (SPL).

Number of participants	71
Age in years (SD)	66.03 (9.15)
**Gender**	
Female	n = 25 mean age 65.68 (9.78)
Male	n = 46 mean age 66.22 (8.89)
**Hearing status**	
Duration of hearing aid use prior to cochlear implantation (SD)	22.12 (14.29)
Duration of severe-to-profound hearing loss in years (SD)	19.98 (14.23)
4-PTA on the better/poorer hearing ear in dB (SD)	80.03 (20.20)/100.03 (11.57)
Unaided monosyllabic speech perception in quiet at 65dB/80dB SPL in% (SD) on the poorer hearing ear	6.79 (12.54)/12.54 (18.45)
Monosyllabic speech perception in quiet in the best aided condition at 65 dB/80 dB SPL in% (SD) on the poorer hearing ear	8.98 (15.73)/16.1 (21.62)

### Audiometric Assessment

Pure-tone thresholds were measured preoperatively for each ear at 0.25–8 kHz in a soundproof booth (DIN EN ISO 8253). Speech testing in quiet was performed pre- and post-operatively by the German Freiburg monosyllabic speech test at 65 and 80 dB by an experienced audiologist preoperatively and 6, 12, and 24 months after implantation. Postoperative speech scores assessed by the Freiburger monosyllabic speech test were performed in CI-only testing condition.

### Neurocognitive Assessment

Subjects underwent a cognitive evaluation preoperatively and 6, 12, and 24 months after cochlear implantation. All the tests were presented only in a visual condition. Before the assessment, the patients were briefed by a professional and given a pretest to familiarize with the test battery. The computer-based neurocognitive assessment tool (ALAcog) consisted of nine subtests covering the following cognitive domains, as previously described by Falkenstein et al. (1999), Völter et al. (2017), and Völter et al. (2018):

- The M3 test assessed attention. Herein, a target letter within distractors had to be correctly identified as fast as possible.
- In the (delayed) recall test, 10 words were presented simultaneously, and they had to be remembered immediately and after 30 min.
- Working memory was assessed by the 2-back and the Operation Span (OSPAN) task. In the 2-back task, subjects had to press each time in case a letter was shown which was identical to the second last. In the Dual-Task Operation Span (OSPAN), equations had to be solved while remembering the letters at the same time.
- To evaluate the inhibitory abilities, the Flanker task was included where the subjects had to react to a target arrow flanked by arrow pointers, above and underneath, pointing in the same (compatible Flanker) or in different directions (incompatible Flanker). The total Flanker score is the difference between the incompatible Flanker (iFlanker) and the compatible Flanker (cFlanker) score.
- The Trail Making Test (TMT) was included to measure the processing speed (TMT A) and mental flexibility (TMT B). In both subtasks, randomly shown items had to be sorted as quickly as possible; in TMT A, numbers from 1 to 26 had and in TMT B, numbers from 1 to 13 and letters from A to M have to be sorted. The total TMT is the difference between TMT B and TMT A. Prior to the TMTs, a motor test was applied, where 26 gray squares are shown on a screen and one of these squares turns into green, in a random order and the participant was asked to click as soon as one square gets green.

A comprehensive set of raw data was created for each subtest including the reaction time and the number of correct and incorrect responses. A total score, the inverse efficiency (IE), was calculated based on the time needed and the number of correct answers given. A lower IE score indicated a better performance. Practice effects were minimized by different test versions.

### Questionnaires

The Nijmegen Cochlear Implant Questionnaire (NCIQ) (Hinderink et al., 2000) was used to evaluate the health-related QoL. It comprised 60 statements with five options to answer, ranging from 0 (not at all) to 100 (very good). The total score was calculated from three domains, which were further divided into 6 subdomains: (1) *physical domain*: (a) basic sound perception, (b) advanced sound perception, and (c) speech production; (2) *psychological domain*: (a) self-esteem; (3) *social domain*: (a) activity limitations and (b) social interactions. A higher score represented a better health-related QoL.

The semi-structured interview Cognitive Reserve Index Questionnaire (CRIq) (Nucci et al., 2012), which measures the CR throughout lifetime, covers information in three subcategories: (1) education, (2) leisure time, and (3) working activity, and also the demographic data. Education was related to the number of school years, occupational training, and other activities, such as learning a new instrument or a language. Leisure time included free-time activities, such as sports and reading. Working activities were classified based on the responsibilities and demands according to the years worked. In the CRIq, participants got 1 point for every completed school year and 0.5 points for every uncompleted school year. Years of vocational training and university years were assessed in the same way. For a doctoral degree, 5 points were added. If other supervised training courses, such as language, chess, or photography courses, were attended, 0.5 points were added for 6 months of attendance for each (Nucci et al., 2012). A total score was calculated by combining the three subdomains adjusted for age. A score less than 70 points represented a low, 70–84 a medium-low, 85–114 a medium, 115–130 a high-medium, and greater than130 a high CR. The results were adjusted for age by correlating the number of years an activity had been carried out by −0.56 for education, by 0.48 for working activity, and by 0.66 for leisure time. Furthermore, age effects have been ruled out by linear models as described by Nucci (Nucci et al., 2012).

Depressive symptoms were questioned by the Geriatric Depression Scale 15 (GDS-15) (Yesavage et al., 1983). A sum score of 15 dichotic statements (yes/no) reflected the severity of depressive symptoms. No depressive symptoms were present with a score between 0 and 5 points, slight to moderate symptoms with a score between 6 and 10 points, and 11 and more points indicated a severe depression.

### Statistical Analysis

Data were tested for distribution first. Whereas QoL, depression, CRI, age, duration of hearing aid use, and duration of deafness, as well as speech perception, were parametric, the cognitive data were mostly non-parametric except the recall test. To ensure consistency, for non-parametric data, the median and the 68% confidence interval, and for parametric data, mean and standard deviation were reported. For all data, rank correlation between two variables was calculated by using Kendall’s τ. For comparison between pre- and post-operative results, the Wilcoxon–Mann–Whitney U test was applied. The individual performance of the subject was compared to the 68% confidence interval prior to implantation. The TMT was calculated with linear models and the rule of proportion, in case the participants were unable to finish the task within the given time. The effect size was calculated using Cohen’s d (d = 0.2–0.4 represented a small, d = 0.5–0.7 a medium, and d ≥ 0.8, a large effect size) for parametric data and after transformation for non-parametric data. Statistical significance was set to *p* < 0.05. In all analyses, the *p*-values were corrected for multiple comparisons using Bonferroni correction to provide statistical accuracy.

Multi-regression analysis was performed to evaluate the possible predictors for cognitive function after data transformation by squaring or calculating the logarithm. For each cognitive subtest (preoperative and postoperative cognitive performance and changes in cognitive performance), an analysis was performed. The CRIq total score, age, gender, and monosyllabic speech perception at 65 dB SPL were used as predictors.

The statistical program used was Medas (Grund, Margetshochheim, Germany).

The study was approved by the ethics institution of the Ruhr- University of Bochum (No. 16-5727-BR). All participants gave their written consent. This study was in line with the Declaration of Helsinki.

## Results

### Cognitive Performance in the Follow-Up After Cochlear Implantation

**FIGURE 1:**
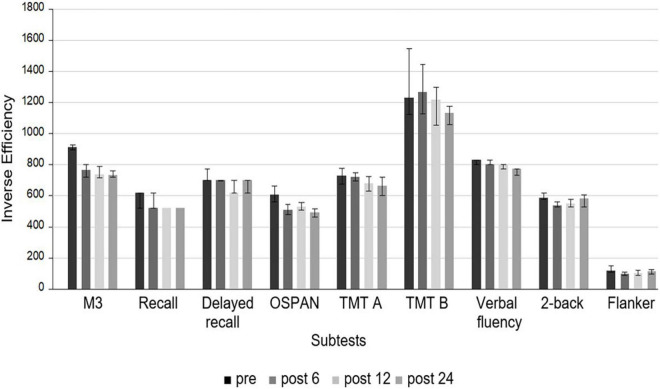
Median of the IE of the neurocognitive subtests at different times (preoperatively, 6, 12, and 24 months after cochlear implantation). A lower IE score indicates a better performance. None of the subtest scores improved between 12 and 24 months after cochlear implantation.

Totally (Figure 1 and Table 2), cognitive functions significantly improved in three out of nine subtests, such as in the M3 (*p* = 0.00004, d = 0.59), OSPAN (*p* = 0.002, d = 0.41), and the Flanker task (*p* = 0.0002, d = 0.34) 6 months after cochlear implantation. Between 6 and 12 months, significant improvements were seen for the recall (*p* = 0.003, d = 0.38) and the verbal fluency task (*p* = 0.0048, d = 0.37). The 2-back performance showed an increase from preoperatively to 6 months postimplantation (*p* = 0.02) and remained stable after 12 months (*p* = 0.9); however, significance was not achieved until 24 months postimplantation (*p* = 0.002, d = 0.32). The incompatible Flanker performance was similar to the total Flanker performance; it significantly improved from preoperatively to 6 months postoperatively (*p* = 0.001, d = 0.2) and remained stable after 12 (*p* = 0.74 and 24 months (*p* = 0.87), whereas the compatible Flanker task remained unchanged over the whole period (each *p* ≥ 0.005).

**TABLE 2:** Median of the inverse efficiency (IE) and 68% confidence interval of the neurocognitive subtests.

Neurocognitive subtest		Median	68% confidence interval		p1 (pre-6)	p2 (6–12)	p3 (12–24)	p4 (pre-24)
M3	pre	909.5	699.47	1,398.23	0.00004*	0.41	0.06	0.00001*
	post 6	764	599.08	1,113.53				
	post 12	737.5	610.31	1,038.16				
	post 24	735	583.67	951.96				
Recall	pre	620	400	700	0.48	0.003*	0.7	0.001*
	post 6	520	260	700				
	post 12	520	260	620				
	post 24	520	260	700				
Delayed recall	pre	700	520	880	0.05	0.022	0.4	0.001*
	post 6	700	456.55	830				
	post 12	620	400	830				
	post 24	700	260	874.71				
OSPAN	pre	604	374.16	901.89	0.002*	0.07	0.97	0.00004*
	post 6	508	358.88	779.39				
	post 12	528	349.73	751.75				
	post 24	492	330.74	757.15				
TMT A	pre	726.5	513.95	1,427.22	0.31	0.21	0.88	0.0005*
	post 6	718	504.47	1,403.75				
	post 12	680.5	473	1,230.49				
	post 24	661.5	472.89	1,242.95				
TMT B	pre	1230	721.89	2,035.69	0.23	0.05	0.37	0.78
	post 6	1264	779.42	2,598.32				
	post 12	1219	697.31	2,008.82				
	post 24	1131.5	800.84	2,156.63				
Verbal fluency	pre	830	735	880	0.07	0.0048*	0.12	<0.00001*
	post 6	800	710.94	855				
	post 12	800	660	855				
	post 24	770	620	830				
2-back	pre	583.5	452.63	977.69	0.02	0.9	0.24	0.002*
	post 6	539	415.31	910.12				
	post 12	546	414.31	771.84				
	post 24	580	393.89	859.05				
Flanker	pre	118	53	352.51	0.0002*	0.38	0.81	0.01
	post 6	96	48.18	163.82				
	post 12	100	38.21	231.25				
	post 24	112	50.79	228.48				

*p1 means comparison between pre- and 6 months, p2 means comparison between 6 and 12 months, p3 means comparison between 12 and 24 months after cochlear implantation, and p4 means comparison between preoperative and 24 month-postoperative performance. A lower IE score indicates a better performance.*

*After Bonferroni correction, the p-value was set to a value < 0.005 and is written in bold.

One year after implantation, improvement reached significance in six out of nine subtests. The M3 (*p* = 0.00001, d = 0.57) and the OSPAN (*p* = 0.00003, d = 0.57) were the neurocognitive subdomains, which improved the most from preoperatively to 12 months postoperatively, whereas recall (*p* = 0.003, d = 0.44), delayed recall (*p* = 0.0002, d = 0.44), and verbal fluency (*p* = 0.003, d = 0.45) showed only a small effect size. No further improvement was observed between 12 and 24 months (each *p* ≥ 0.06).

After 2 years (Table 2), cognitive performance in almost all subtests was significantly better than pre-implantation (each *p* ≤ 0.002) except for the total Flanker (*p* = 0.01) and the total TMT (*p* = 0.04). Whereas TMT A did not improve earlier than after 24 months (*p* = 0.0005, d = 0.37), TMT B remained unchanged between preimplantation and 24 months (*p* = 0.78) which resulted in a decrease in the total TMT score after 24 months (*p* = 0.04). This was independent of the motor functions, which did not change during the follow-up period (each *p* ≤ 0.22).

Based on the individual performance of the subjects, 12 out of 71 subjects performed worse than the 68% confidence interval in three or even more subtests preoperatively, and in only three out of 71 subjects one year after cochlear implantation. One subject achieved comparable scores in three subtests, another subject improved his/her performance from initially 4 to 3 poorly done tests, and another individual could not keep up his/her performance in 4 instead of 3 subtests.

Analyzing the subtests showed that most of the initial poor test results improved and reached a level within or even above the range of 68% two years after cochlear implantation. This was true for the M3 in 7 of 7 cases, for the recall in 6 of 7, the delayed recall in 7 of 9, the OSPAN in 8 of 11 patients, for the Flanker in 7 of 10, and for the verbal fluency in all 8 subjects. However, the 2-back test improved only in 5 of 10 and the TMT A in 3 of 10, and the TMT B in 3 of 9 subtests.

Between the first and the second year after cochlear implantation, the performance remained stable in 3 or more tests in 70 and in 5 or more tests in 63 individuals. Among 5 subjects, the performance decreased in more than 2 tests and in one subject in more than 3 tests. Four individuals had better results in more than 3 tests after 12 months.

Cognitive function positively correlated with age, both pre- and post-operatively. Prior to CI, TMT A and B were poorer with increasing age (*p* = 0.00047 and *p* = 0.0004). Twelve months after implantation, recall and TMT A (*p* = 0.0015 and *p* = 0.0046), and 2 years after operation, M3 and TMT A correlated with each other (*p* = 0.00012 and *p* = 0.0015). Improvement in cognitive functions was independent of age in all different subtests and at each time (each *p* ≥ 0.07). Speech perception in quiet at 65 dB and at 80 dB was not correlated with cognitive function at any time point (each *p* ≥ 0.005). Furthermore, improvement in speech perception did not correlate with any cognitive subtests (each *p* ≥ 0.02). None of the subtests differed according to gender.

### Cognitive Reserve Index

The mean overall cognitive reserve index (CRI) in the 68 subjects who answered this questionnaire was 111.35 (SD = 14.55), 99.73 (SD = 15.67) for the subcategory of working activity, 118.69 (SD = 19.66) for leisure time activities, and 107.15 (SD = 11.04) for the educational background.

The overall CRI score was similar between men and women [111.20 (SD = 13.65) and 112.42 (SD = 14.99); p = 0.52]. Women [122.94 (SD = 20.85)] were more involved in leisure time activities than men [110.56 (SD = 15.91); *p* = 0.003] and men achieved a higher working score [107.68 (SD = 11.56) and 97.73 (SD = 16.01); *p* = 0.003]. Age positively correlated with the CRI score (τ = 0.27, *p* = 0.001) and with the subdomains of education (τ = 0.24, *p* = 0.004) and leisure time (τ = 0.302, *p* = 0.0003), but did not correlate with the subdomain of working activity (τ = 0.003, *p* = 0.97).

**FIGURE 2:**
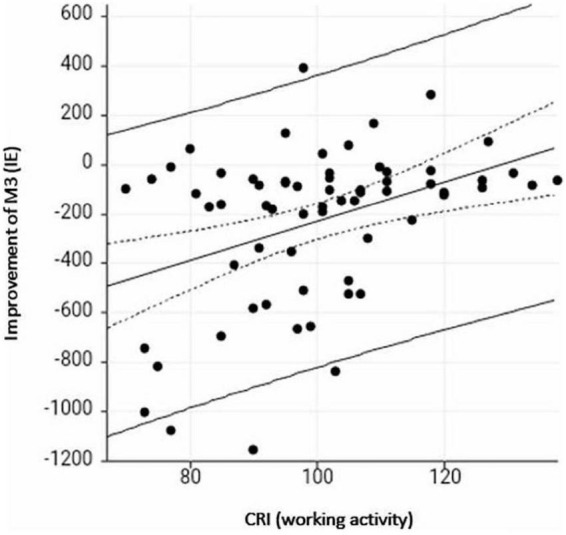
Scatterplot of CRI working activity and improvement in the attentional task (inverse efficiency) from pre- to 24 months post implantation. A lower score on the y-axis (M3 test) indicates greater improvement, a higher score on the x-axis (CRI-working activity) indicates a higher cognitive reserve index (CRI).

Subjects with a higher total CRI achieved better preoperative cognitive results in 2 out of 9 tests (OSPAN τ = −0.33, *p* = 0.00008 and verbal fluency task: τ = −0.023, *p* = 0.006). The highest preoperative correlation was found between cognitive functions and the CRI category of working activity. No correlation was detected with leisure time activities and education (each *p* ≥ 0.005). Twelve months post-implantation, a better total CRI was associated with a better IE of the TMT B (ττ = −0.25, *p* = 0.003). After 24 months, none of the cognitive subtests correlated with the CRI total score (each *p* ≥ 0.005) or any subcategory, except verbal fluency with the subcategory of leisure time activities (τ = −0.24; *p* = 0.003). Improvement in cognitive functions after 24 months was greater for the attentional task M3 in case of a lower score in the working activity domain (τ = 0.24, p = 0.003) (Figure 2).

Improvement in the QoL did not depend on the CRI (total score or subdomains) (each *p* ≥ 0.14). Preoperative and 12 months postoperative speech perception at 65 dB in quiet was independent of the CRI (each *p* ≥ 0.08). The only correlation that was found was between the subdomain of the working activity and the monosyllabic speech perception at 65 dB (τ = 0.24, *p* = 0.004) and 80dB (τ = 0.26, *p* = 0.002) after 2 years, with a better speech perception in case of a better working activity score. The other subdomains did not show any correlation (each *p* ≥ 0.06).

Multiple regression analysis showed that the total CRI was the most important predictor for preoperative cognitive functions in 4 out of 9 tests (M3, Recall, OSPAN, verbal fluency; each p ≤ 0.005), followed by age in 3 out of 9 cognitive subtests (delayed recall, TMT A, TMT B, Flanker; each *p* ≤ 0.0005). Two years after implantation, age was the most important predictor in 5 out of 9 subtests (M3, Flanker, TMT A and B, and Recall).

### Depression

In 59 subjects, the depressive assessment was performed pre- and post-operatively. The mean level of depressive symptoms was 2.65 (SD = 2.6). Fifty-one subjects reported not to have any depressive symptoms at all (GDS < 6), 8 subjects reported mild affective disorders (GDS: 6–10). None of the subjects included suffered from major depression. Post-implantation, the level of depressive symptoms significantly decreased to 1.96 (SD = 2.19) (*p* = 0.01). The level of preoperative depressive symptoms was not correlated with preoperative cognitive function (each *p* ≥ 0.01), but with performance in the verbal fluency task after 12 months (τ = 0.23, *p* = 0.0048). No significant correlation was found between postoperative depressive symptoms and postoperative cognitive skills (each *p* ≥ 0.01). In line with that, the decrease of depressive symptoms did not correlate with the change in any cognitive subtest (each *p* ≥ 0.04).

### Audiometric Performance After Cochlear Implantation

**FIGURE 3:**
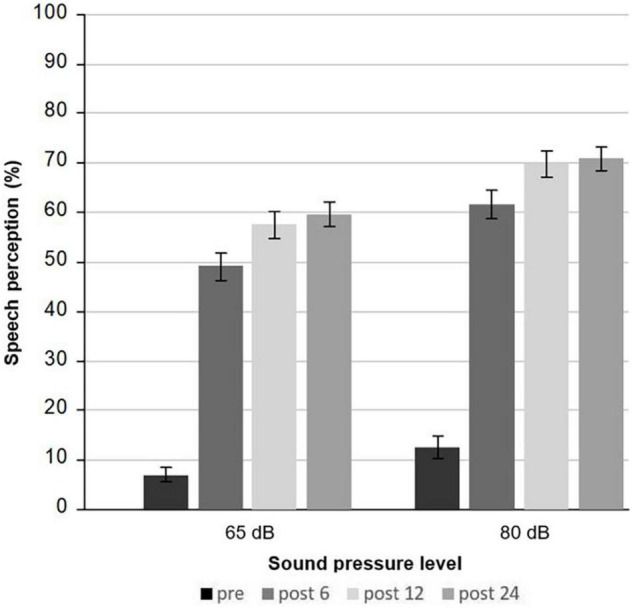
Mean monosyllabic speech understanding at 65 and 80 dB assessed by the Freiburg monosyllabic speech test preoperatively, and 6, 12, and 24 months after cochlear implantation.

Monosyllabic speech perception at 65 dB on the implanted ear significantly improved with a large effect size from pre- to 6 months postimplantation [from 6.96% (SD = 12.5) to 49.06% (SD = 22.92); *p* < 0.0001, d = −1.9] (Figure 3). Between 6 and 12 months, further improvement could be detected (*p* = 0.00009, d = −0.54), whereas after 1 year, speech perception remained stable (*p* = 0.45). The same was true for speech perception at 80 dB. Patients improved from 12.54% (SD = 18.30) to 61.59% (SD = 23.6) (*p* < 0.0001, d = −2.02) after 6 months, to 69.85% (SD = 21.95) between 6 and 12 months after implantation (*p* = 0.00007, d = −0.56). No further benefit was observed between 12 and 24 months (*p* = 0.88). After 2 years, 59.56% (SD = 20.79) of the monosyllabic words were correctly understood at 65 dB and 70.92% (SD = 19.52) at 80 dB. Speech perception did not correlate with depressive score at any time point (each *p* ≥ 0.04).

### Changes in Health-Related QoL After Cochlear Implantation

Data from the Nijmegen Cochlear Implant Questionnaire were available for 69 subjects before and 6, 12, and 24 months after implantation (Table 3). Before implantation, the category social with the two subcategories of activity limitation and social interaction was judged to be mostly impaired, followed by the category, advanced sound perception.

**TABLE 3:** Mean score of the Nijmegen Cochlear Implant Questionnaire.

Nijmegen subscores		Mean	Standard deviation	p1 (pre-6)	p2 (6–12)	p3 (12–24)	p4 (pre-24)
Basic sound perception	pre	48.62	22.54	<0.00001*	0.47	0.56	<0.00001*
	post 6	70.48	17.53				
	post 12	71.11	17.59				
	post 24	74.22	17.61				
Advanced sound perception	pre	45.93	23.26	<0.00001*	0.17	0.69	<0.00001*
	post 6	62.82	17.59				
	post 12	65.11	19.84				
	post 24	66.70	17.15				
Speech production	pre	65.99	19.22	<0.00001*	0.44	0.37	<0.00001*
	post 6	76.99	16.25				
	post 12	79.34	15.41				
	post 24	79.01	15.07				
Self esteem	pre	47.01	18.27	<0.00001*	0.04	0.22	<0.00001*
	post 6	58.42	16.18				
	post 12	62.35	16.0				
	post 24	64.33	14.95				
Activity limitations	pre	43.62	21.4	<0.00001*	0.33	0.17	<0.00001*
	post 6	59.83	18.48				
	post 12	62.44	21.09				
	post 24	65.37	16.94				
Social interactions	pre	45.18	21.27	<0.00001*	0.06	0.42	<0.00001*
	post 6	61.56	17.01				
	post 12	64.66	20.21				
	post 24	66.9	16.89				
Physical	pre	53.69	18.62	<0.00001*	0.3	0.67	<0.00001*
	post 6	69.9	14.13				
	post 12	71.89	15.18				
	post 24	73.31	13.99				
Social	pre	44.40	20.32	<0.00001*	0.12	0.33	<0.00001*
	post 6	60.7	16.67				
	post 12	63.55	19.84				
	post 24	66.13	16.29				
**Total score**	pre	49.57	17.00	<0.00001*	0.1	0.4	<0.00001*
	post 6	64.71	13.73				
	post 12	67.34	15.23				
	post 24	69.24	12.85				

The value p1 means p-value for the comparison between pre- and 6 months, p2 means p-value for the comparison between 6 and 12 months, p3 means p-value for the comparison between 12 and 24 months after cochlear implantation, and p4 means p-value for the comparison between preoperative performance and 24 months postoperatively. *After Bonferroni correction, the p-value was set to < 0.005 and is written in bold.

**FIGURE 4:**
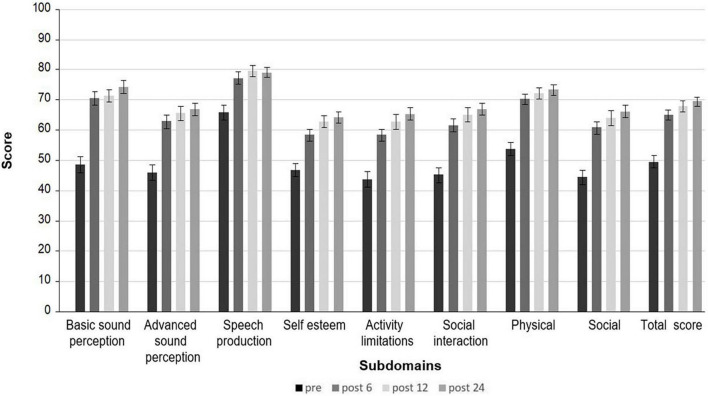
Mean score of the Nijmegen Cochlear Implant Questionnaire. Preoperatively and 6, 12, and 24 months after cochlear implantation.

After 6 months post-implantation, subjects significantly improved with a large effect size in the total score (*p* < 0.00001; d = −1.07) and in the subdomains (each *p* < 0.00001). There was no further improvement between 6 and 12 months after Bonferroni correction with *p* < 0.005 (each *p* ≥ 0.04) (Figure 4). The greatest improvement 1 year after implantation with 22.49 additional points was seen for the category of basic sound perception (d = −1.08). The subcategory of social interaction improved by 19.48 extra points (d = −1.12) and advanced sound perception by 19.18 (d = −1.0). Results remained stable up to 24 months after cochlear implantation (each *p* ≥ 0.17). Depression was highly correlated with the QoL pre- and post-operatively. Before cochlear implantation 8 out of 9 and postoperatively 7 out of 9 Nijmegen subcategories were rated lower in case of a higher GDS-15 score. Further, improvement in QoL after 24 months came along with an improvement in the depressive symptoms (τ = −0.32, *p* = 0.0009).

No correlation was found between health-related QoL and cognitive functioning neither pre-, nor postoperatively, after Bonferroni correction (each *p* ≥ 0.005).

## Discussion

To the best of our knowledge, the present study is one of the first to evaluate a broad spectrum of different cognitive subdomains for a follow-up of 2 years in a large population in a single-center study with multiple fixed time points. So far, only a few single-center studies have analyzed the long-term effects of cochlear implantation after one year (Sarant et al., 2019; Huber et al., 2020). Few studies repeatedly evaluated speech perception, cognitive domains, and health-related QoL in the same participants to study the impact of auditory restoration on cognition (Mosnier et al., 2015, 2018; Völter et al., 2018, 2020b; Sarant et al., 2019).

Auditory rehabilitation by cochlear implantation significantly improved the neurocognitive functions of people with hearing impairment; however, enhancement differed for the neurocognitive subtests. Whereas attentional driven domains, such as attention, inhibition, and working memory already improved after 6 months, mainly memory-based tests as short- and long-term memory and verbal fluency first improved after 12 months.

Subjects with hearing impairment and a poor preoperative cognitive performance showed a greater benefit from cochlear implantation than those with better cognitive skills. This has already been reported by Mosnier et al. (2015) in 30 out of 37 subjects with preoperative abnormal scores in two or more of the cognitive tests (Mosnier et al., 2015) and by Zhan et al. (2020) in 19 CI users with a medium to large effect size (Zhan et al., 2020).

Although the impact of hearing loss on psychosocial well-being is already known and the improvement after cochlear implantation has been described in various studies (Olze et al., 2011; Mosnier et al., 2015; Brüggemann et al., 2017; Lawrence et al., 2020), its influence on cognitive performance has been analyzed only by Huber et al. (2020), who described that people suffering from depressive symptoms required significantly more time to complete the TMT B task, and by Castiglione et al. (2016), who elucidated a negative correlation between the Geriatric Depression score and the performance on the Montreal Cognitive Assessment in a sample of 15 CI subjects post-implantation (Castiglione et al., 2016). However, in the present study, the correlation between depressive symptoms and cognitive functions was small and only significant in verbal fluency.

The overall CR score positively correlated with preoperative cognitive performance in working memory. Subitem analysis revealed that the subdomain of working memory came along with better results in the M3, the verbal fluency, and in the Flanker tasks. In contrast, subjects with a lower CRI working activity subscore improved the most in the attentional task. This has also been observed by Chan et al. (2018) who analyzed the cognitive ability and brain structure in subjects with low and high mid-life activities (Chan et al., 2018). So far, the impact of socioeconomic background in this context is sparse in literature and data have been reported only with regard to the educational background. Sarant et al. (2019) reported in a study on 59 CI candidates that executive functions as assessed by the Groton Maze learning test highly correlated with the subject’s educational background, but improvement in the latter was only significant after 18 months for men without tertiary education, whereas in all other participants, it remained stable. However, it has been shown in a longitudinal cohort study in 2,899 subjects aged 77.8 who underwent annual cognitive testing, that education only correlated with the initial level of cognitive function but not with the rate of cognitive decline in the composite measures of global cognition, episodic memory, and perceptual speed (Wilson et al., 2019).

Notably, the greatest improvement in cognitive performance was within the first 6 months after cochlear implantation. Between 12 and 24 months, cognition remained stable and the pattern of enhancement in the cognitive function was similar to the pattern of improvement in speech perception and in QoL, even if the effect size in cognition was smaller than in the speech domain. In total, a significant improvement in almost all the studied cognitive subdomains was detected from pre- to 24-months postimplantation. This is remarkable if you keep in mind that a decrease in cognitive performance, mainly in fluid intelligence, is regarded as a part of the physiological aging process (Salthouse, 2009; Diamond, 2013; Whitley et al., 2016).

Therefore, the present data may support the cognitive load theory hypothesis. Given the limited capacity of cognitive resource, a decrease in the listening effort by CI might have re-allocated the cognitive resource to other cognitive processes and thereby enhanced the overall cognitive functions. However, no correlation was observed among the improvement in speech perception, cognitive abilities, and QoL in the present study. This has already been observed by others. Although improvement in QoL and speech perception have been described up to and beyond 2 years, the improvement increased the most within the first 6 months of the device use (Olze et al., 2011; Lenarz et al., 2012; Völter et al., 2018; Andries et al., 2021); only a weak association between health-related QoL and speech perception in CI users has been described in previous studies (Moberly et al., 2017; Vasil et al., 2020). In a meta-analysis by McRackan et al. (2017) covering 13 articles with 715 subjects, correlation between speech perception measures, such as word or sentence recognition in quiet and sentence recognition in noise, and QoL in total or in the different subscores was only low or even neglectable (McRackan et al., 2017).

Further, an association of auditory and cognitive performance following cochlear implantation has been rarely reported (Cosetti et al., 2016; Wazen et al., 2020; Zhan et al., 2020; Knopke et al., 2021). Zhan et al. found only a correlation of sentence recognition in quiet after 6 months with the incompatible Stroop and the Symbol Span test, but not for the Digits or Object Span test (Zhan et al., 2020). Huber et al. reported a correlation of an improvement in the Clock Drawing test with monosyllabic and sentence recognition in quiet 3 months post-implantation, but not after 12 months (Huber et al., 2021). Mosnier et al. found no association between speech perception in quiet or in noise and in cognitive measures (Mosnier et al., 2018). This was also true in the study by Knopke et al. that analyzed speech perception in quiet and in noise and in the Wechsler Adult Intelligence Score (WAIS-IV scores) (Knopke et al., 2021).

On the other hand, cochlear implantation might also have an indirect effect on cognition. Considering cochlear implantation as a proactive plan of the subject with hearing impairment to deal with a disease, it may thereby be a strategy to slow down the age-related cognitive decline. Having a purpose in life has been shown to be associated with the reduced risk of Alzheimer’s disease in a longitudinal epidemiologic clinicopathologic study in 246 older subjects by Boyle et al. including cognitive evaluations and brain autopsy, even after controlling potentially confounding variables (Boyle et al., 2012).

Furthermore, one has to keep in mind that cochlear implantation is embedded in a complex rehabilitation setting with multiple appointments at the CI center including audio processor fitting and auditory training in the first months after implantation, and therefore entail enhanced opportunities to engage in social and cognitive stimulation. The role of an enriched environment to stimulate the plasticity of the brain in the elderly and thereby to counteract the age-dependent decline of cognitive performance has already been described in the sixties in animal studies and in humans (Diamond et al., 1964; Eisenstein et al., 2021). Activities that combine physical activity, social interaction, sensory and cognitive stimulation have been shown to be an environmental enrichment leading to an improved performance also in non-trained tasks (Li et al., 2011; Bavelier et al., 2012). Especially for individuals without a college degree, increased cognitive engagement in older age, such as reading, doing word games, and attending educational courses, is important for reducing the decline in executive functions. This emphasizes the importance of promoting and encouraging increased engagement especially among those with lower educational attainment who generally are at greater risk to cognitive decline (Stieger and Lachman, 2021). Furthermore, social network or frequency of contacts, mainly with friends might promote cognitive health and reduce the risk of dementia (Sommerlad et al., 2019; Röhr et al., 2020). Over time, successful rehabilitation after cochlear implantation might also change the social interaction and free leisure time activities (Hawthorne et al., 2004; Nijmeijer et al., 2021) and thereby increase the CR. However, the study period of two years might be too short to report on these changes; further investigations, including the assessment of the CR in the follow-up after the restoration of hearing loss, should be performed in the future.

Another limitation of the present study is that a control group is missing, due to ethical reasons. This is a weakness in most studies in this field (Miller et al., 2015; Dawes, 2019; Moberly et al., 2019). Some studies, such as one by Jayakody et al., did a comparison of CI candidates and CI recipients, although ideal matching is challenging (Jayakody et al., 2017; Huber et al., 2020). The study by Mertens et al. was the only one that enrolled a control group of CI candidates matched to CI recipients in terms of gender, age, formal education, cognitive functioning, and residual hearing. But even in this high-quality study, the sample size was small and inhomogeneous and bias cannot be ruled out (Mertens et al., 2021). Another approach was applied by Huber et al. (2021). Twenty-nine adult subjects aged 60–80 years scheduled for cochlear implantation and an age- and education-matched control group of normal-hearing subjects were enrolled in this study. However, a clinical intervention group and a healthy untreated control group might be difficult to compare. Therefore, our approach to study cognition in the same subject in the longitudinal follow-up of 2 years in a single center and with fixed appointments seems reasonable. However, 5- or even 10-year data might be important and should be looked on in further studies.

Whether cochlear implantation also has a positive effect on subjects with cognitive dysfunction cannot be answered as subjects with severe cognitive impairment were excluded in the present study. So far, cognitive changes after cochlear implantation in people with cognitive impairment have been studied in detail only by few (Mosnier et al., 2018; Gurgel et al., 2021). Half of the 38 subjects with an MCI remained stable, 10 improved, and only two developed dementia, whereas 12 out of 54 with preoperative normal cognitive functions suffered from MCI 7 years after implantation in a study done by Mosnier et al. (2018).

## Conclusion

Auditory rehabilitation by cochlear implantation has a positive impact on auditory functions, QoL, and neurocognitive functioning. The present study clearly showed that cognition significantly improves after cochlear implantation, mostly 6 months after the primary audio processor fitting. However, there was no correlation between cognitive performance and the hearing level or QoL. Therefore, cochlear implantation might be considered a multifactorial active treatment that creates an enriched environment stimulating the plasticity of the brain, especially in subjects with poor preoperative performance and a low cognitive reserve.

## Data Availability Statement

The original contributions presented in the study are included in the article/supplementary material, further inquiries can be directed to the corresponding author.

## Ethics Statement

The studies involving human participants were reviewed and approved by Ethics Institution of the Ruhr- University of Bochum, Germany (No. 16-5727-BR). The patients/participants provided their written informed consent to participate in this study.

## Author Contributions

CV and JT designed the study. LG selected the subjects. MB collected a part of the data. CV and LG analyzed and evaluated the data. CV and LG wrote the manuscript with contributions and critical feedback from all authors. SD supervised the project.

## Conflict of Interest

The research project of the present manuscript did not receive any funds from any company. CV, JT, and SD have received reimbursement of scientific meeting participation fees and accommodation expenses as well as honoraria for preparing continuing medical education events and funding for other research projects from MED-EL. The remaining authors declare that the research was conducted in the absence of any commercial or financial relationships that could be construed as a potential conflict of interest.

## Publisher’s Note

All claims expressed in this article are solely those of the authors and do not necessarily represent those of their affiliated organizations, or those of the publisher, the editors and the reviewers. Any product that may be evaluated in this article, or claim that may be made by its manufacturer, is not guaranteed or endorsed by the publisher.
